# Fate of micronuclei and micronucleated cells after treatment of HeLa cells with different genotoxic agents

**DOI:** 10.1007/s00204-022-03433-9

**Published:** 2022-12-23

**Authors:** Hauke Reimann, Helga Stopper, Henning Hintzsche

**Affiliations:** 1 Institute of Pharmacology and Toxicology, University of Würzburg, Würzburg, Germany; 2 Bavarian Health and Food Safety Authority, Erlangen, Germany; 3 Department of Food Safety, University of Bonn, Bonn, Germany

**Keywords:** Fate of micronuclei, Chromosomal DNA damage, Methyl methanesulfonate, Vinblastine, Ionizing radiation, Doxorubicin, Tert-butyl hydroperoxide

## Abstract

Although micronuclei are well-known biomarkers of genotoxic damage, the biological consequences of micronucleus induction are only poorly understood. To further elucidate these consequences, HeLa cells stably expressing histone 2B coupled with green fluorescent protein were used for long-term live cell imaging to investigate the fate of micronuclei and micronucleated cells after treatment of cells with various genotoxic agents (doxorubicin (20, 30 and nM), tert-butyl hydroperoxide (tBHP, 50, 100 and 150 µM), radiation (0.5, 1 and 2 Gy), methyl methanesulfonate (MMS, 20, 25 and 30 µg/ml) and vinblastine (1, 2 and 3 nM)). Most micronuclei persist for multiple cell cycles or reincorporate while micronucleated cells were more prone to cell death, senescence and fatal mitotic errors compared to non-micronucleated cells, which is consistent with previous studies using etoposide. No clear substance-related effects on the fate of micronuclei and micronucleated cells were observed. To further investigate the fate of micronuclei, extrusion of micronuclei was studied with treatments reported as inducing the extrusion of micronuclei. Since extrusion was not observed in HeLa cells, the relevance of extrusion of micronuclei remains unclear. In addition, degradation of micronuclei was analysed via immunostaining of γH2AX, which demonstrated a high level of DNA damage in micronuclei compared to the main nuclei. Furthermore, transduction with two reporter genes (LC3B-dsRed and LaminB1-dsRed) was conducted followed by long-term live cell imaging. While autophagy marker LC3B was not associated with micronuclei, Lamin B1 was found in approximately 50% of all micronuclei. While degradation of micronuclei was not observed to be a frequent fate of micronuclei, the results show impaired stability of DNA and micronuclear envelope indicating rupture of micronuclei as a pre-step to chromothripsis.

**Supplementary Information:**

The online version contains supplementary material available at 10.1007/s00204-022-03433-9.

## Introduction

During mitosis, two identical daughter cells normally arise after cell division. In some cases, daughter cells are accompanied by one or multiple small nuclear bodies called micronuclei. Like the main nucleus, micronuclei consist of chromatin surrounded by a (micro-) nuclear membrane. However, it is not entirely clear, how stable the micronuclear membrane is, as envelope rupture and impaired ability to replicate, transcribe or repair the DNA inside a micronucleus have been observed (Hatch et al. 2013). Several types of micronuclei can be distinguished based on their content: Micronuclei can contain whole chromosomes, chromosomal fragments or extrachromosomal amplified DNA, so-called double minutes (DM) (Terradas et al. 2010). They can originate e.g. from microtubule spindle disturbances or disorders of a centromere region causing whole chromosome micronuclei derived from lagging chromosomes, while DNA double strand breaks or breakage of chromosomal bridges primarily result in micronuclei with chromosomal fragments (Hoffelder et al. 2004; Kisurina-Evgenieva et al. 2016). DM are localised at the periphery of the nucleus during mitosis but unrepaired DNA damage may cause the formation of DM-aggregates, which lag behind and form micronuclei (Shimizu 2011).

Micronuclei have been used for decades as biomarker for the assessment of genotoxicity of pharmaceuticals, pesticides and other chemicals (Fenech 2007; OECD 2016). In addition, the presence of micronuclei in peripheral blood lymphocytes is linked to cancer risk (Bonassi et al. 2007). Despite this long-term application, the fate of micronuclei and micronucleated cells is only poorly understood, which makes assessment of the biological relevance of micronuclei for the organism, e.g., for tumour formation, difficult (Guo et al. 2019). However, several possible fates have been proposed for micronuclei: Degradation of the micronucleus, extrusion of the micronucleus into the extracellular space, apoptosis triggered by the micronucleus, persistence of the micronucleus and micronucleated cell and reintegration of the micronucleus during one of the next mitoses (Stopper and Hintzsche 2019). While apoptosis with the loss of both the cell and the micronucleus could reduce potential harm caused by micronuclei, loss of the genetic information of the micronuclear DNA via degradation or extrusion as well as maintaining micronuclei by persistence or reintegration could promote tumour development. Another mechanism associated with micronuclei is chromothripsis. Chromothripsis describes the massive rearrangement within a chromosome, while other parts of the genome remain unaffected. Micronuclei pose one explanation, why only certain genomic regions are affected by rearrangements and additional DNA damage (Crasta et al. 2012).

We recently observed in etoposide-treated cells, that persistence and reintegration occurred regularly, while degradation and extrusion of micronuclei were only rarely or never seen. Furthermore, the cell death rate was significantly increased in micronucleated cells (Reimann et al. 2020a, b). Interestingly, these findings were not completely in accordance with the available literature data. For example, degradation of micronuclear chromatin was proposed based on the observation of γH2AX foci (Terradas et al. 2009). Also, co-localisation of micronuclei with autophagic proteins was observed and was suggested to demonstrate an elimination mechanism (Rello-Varona et al. 2012). Apart from degradation, the lack of extrusion in our previous observations is in contrast to other results, e.g., after treatment with cytochalasin B (Nito et al. 1988). On the other hand, persistence and reincorporation are consistently reported in the literature, particularly in live-imaging studies, even though there is some quantitative variability (Huang et al. 2011; Soto et al. 2018).

The aim of the current study was to clarify if the fate of micronuclei and micronucleated cells is substance-dependent, i.e., whether previous findings might be specific for etoposide or topoisomerase inhibitors or whether these observations can be generalized independently of the type of micronucleus induction. For this reason, we conducted live cell imaging using HeLa-H2B-GFP cells after administration with different doses of doxorubicin, tert-Butyl hydroperoxide (tBHP), radiation, methyl methanesulfonate (MMS) and vinblastine. These agents were chosen because they induce DNA damage via different mechanisms, in order to cover the different types of micronucleus induction. Degradation of micronuclei was investigated with γH2AX-staining in HeLa-H2B-GFP cells and with live imaging using HeLa-H2B-GFP cells carrying reporter genes with membrane integrity marker Lamin B1 and autophagy marker LC3B. Furthermore, extrusion induction was attempted via induction under special treatment conditions.

## Materials and methods

### Treatment and cell culture conditions

HeLa cells stably transfected with GFP-tagged histone 2B were used for most experiments and were provided by Noriaki Shimizu (Graduate School of Integrated Sciences for Life, Hiroshima University, Japan). Transfection protocol and authentication of cell line was published before (Kanda et al. 1998). Cell culture was conducted at 37 °C and 5% CO_2_ using DMEM High Glucose Medium without phenol red (Sigma–Aldrich) supplemented with 10% FCS (Merck), 2 mM L-glutamine (Sigma–Aldrich), 100 µg/ml streptomycin (Sigma–Aldrich), 100 U/ml penicillin (Sigma–Aldrich), 1 mM sodium pyruvate (Sigma–Aldrich) and 25 mM HEPES (Sigma–Aldrich). Vinblastine and doxorubicin (Teva) were dissolved in water and applied for 4 h, tBHP (Sigma–Aldrich) was dissolved in water as well and applied for 30 min. MMS (Sigma–Aldrich) was dissolved in DMSO and applied for 4 h. Radiation was conducted on a CP160 (Faxitron) on petri dishes using X-rays. Cytochalasin B (Sigma–Aldrich) was dissolved in DMSO and used alone or in combination with etoposide (Teva), that was dissolved in DMSO as well and applied for 3 h. Hydroxyurea (Sigma–Aldrich) was dissolved in water and applied for 72 h before imaging or for the whole observation period during live imaging.

### Cytokinesis-block micronucleus test

Cytokinesis-block micronucleus test with fixed cells was applied to find appropriate doses for micronucleus induction without relevant cytotoxicity. After treatment, medium was changed and 1.5 µg/ml cytochalasin B (Sigma–Aldrich) was added for 22–24 h. Next, cells were trypsinised and cytospinned onto slides, which were fixed for at least 2 h in − 20 °C methanol. After fixation, staining was conducted with GelGreen (Biotium). Micronuclei were scored in binuclear cells as described before (Fenech 2007). Mono-, bi- and multinuclear as well as mitotic and apoptotic cells were evaluated in 1,000 cells, micronuclei were scored in 1,000 binuclear cells. Each analysis was performed on two slides.

Cytokinesis-block proliferation index (CBPI) was calculated with the following formula:

$$\frac{1*\left(\mathrm{No}.\,\mathrm{ of\, mononuclear\, cells}\right)+2*\left(\mathrm{No}.],\mathrm{ of\, binuclear\, cells}\right)+3*(\mathrm{No}.\,\mathrm{ of\, multinuclear\, cells})}{\left(\mathrm{No}.\,\mathrm{ of\, mononuclear\, cells}\right)+\left(\mathrm{No}.\,\mathrm{ of\, binuclear\, cells}\right)+(\mathrm{No}.\,\mathrm{ of\, multinuclear\, cells})}$$

### Construction of expression vectors with Lamin B1-dsRed/LC3B-dsRed and transduction into HeLa-H2B-GFP cells

RNA of HeLa-H2B-GFP cells was isolated, cDNA synthesized and sequences of interest (Lamin B1 or LC3B) amplified with PCR using appropriate primers generating an overhang on both ends. Using gel electrophoresis, PCR products were isolated and purification was conducted with gel extraction. With these products, construction of expression vector was performed with linearised pHAGE-CMV-dsRED-vector carrying an ampicillin resistance gene using Gibson Assembly Master Mix (New England Biolabs). Expression vectors were transformed to 5-alpha Competent *E. coli* (High Efficiency, New England Biolabs) and transformed colonies were picked and cultivated, DNA was isolated and plasmids were sequenced to examine expression vectors. HEK293 cells were transfected with expression vectors, envelope (pMD2.G) and packaging vectors (psPAX2) to produce lentiviruses, which were collected for several days. After that, lentiviruses were filtrated and ultracentrifuged with 20% sucrose. After removing supernatant, viruses were resuspended and used to transduce HeLa-H2B-GFP cells and to generate HeLa-H2B-GFP-Lamin B1-dsRed and HeLa-H2B-GFP-LC3B-dsRed cells.

### Live imaging

All imaging experiments were conducted on glass bottom plates (Cellvis). After treatment of HeLa-H2B-GFP cells, medium was changed and treated cells were cultivated for 24 h to allow for micronucleus induction. Only after radiation, cells were trypsinised from petri dishes and transferred to a glass bottom plate. On the following day, the cell plate was mounted in the live-imaging chamber of a Nikon Ti-S fluorescence microscope equipped with a motorized table and an incubation envelope (Okolab). Temperature, CO_2_ and humidity were maintained. The software NIS-Elements Advanced Research version 5.10.01 (Nikon) was used to control the system. Only cells with micronuclei were selected and positions saved for tracking these cells. Every 10 min images were taken for 96 h. Selected micronucleated cells were termed F0, the following generations were termed F1–F5. Images were taken with an Andor Luca S (Andor Technology) camera with ND 64 and exposure time of 100 ms without binning. 40 × 0.75 NA objective (Nikon) was used. Similar experiments with HeLa-H2B-GFP-Lamin B1-dsRed and HeLa-H2B-GFP-LC3B-dsRed cells were conducted after treatment with etoposide or tBHP at ND 8, 300-ms exposure time and 2 × 2-Binning using z-stacks with 5 levels in 2.5 µm steps around focal plane.

### Evaluation

Image analysis was conducted with Fiji version 1.52. Appearance and timing of mitosis, cell death or cell quiescence was noted. Shrinking and increasing signal strength of the nucleus or swelling of the nucleus with reduced signal intensity was defined as cell death. Furthermore, the duration of mitosis and interphase was noted. Mitotic errors in form of cell death during mitosis, mitosis after fusion of two nuclei during mitosis (probably in multinuclear cells) and multipolar mitosis were quantified. Cells were excluded from the evaluation, when they left the field of view or could not be identified clearly.

The analysis included presence of micronuclei in a cell as well as special events like extrusion, degradation, persistence and degradation. Since tracking of individual micronuclei during mitosis was not possible, evaluation was conducted for each generation separately. Persistence was assumed, when a micronucleus was observed before and after mitosis, while reincorporation was assumed when micronuclei were only visible before mitosis. Micronucleus formation was noted, when micronuclei appeared only after mitosis. 30 micronucleated cells and daughter cells were analysed per experiment. All experiments were conducted five times. Data from control cells, non-micronucleated and micronucleated cells, were obtained from previous experiments (Reimann et al. 2020a, b).

To assess the size of the micronuclei, the area of the micronuclei after treatments and control was measured on images from live imaging after micronucleus formation using Fiji version 1.5.2. Micronuclei with areas smaller than the median of 4.69 µm^2^ were assessed as small, and micronuclei with larger areas as large. Micronuclei from three experiments per treatment or control were analysed.

During experiments with HeLa-H2B-GFP-Lamin B1-dsRed and HeLa-H2B-GFP-LC3B-dsRed cells, co-localisation of a dsRed-signal with micronuclei was evaluated in 25 micronucleated cells (and respective daughter cells) for each of the four experiments. For investigation of a co-localisation of LC3B with micronuclei, only a constant signal over multiple images was considered in order to exclude coincidental co-localisation for a short period, which is assumed to be without biological relevance.

### γH2AX-immunostaining

HeLa-H2B-GFP cells were treated with high doses of each genotoxic agent as before. In addition, the same protocol with control cells without treatment was conducted. Cells were trypsinised and cytospinned onto slides either immediately or after each of the following five days. Fixation was conducted with 4% paraformaldehyde for 10 min followed by two washing steps. Permeabilization was performed with 1% Triton X-100 for 15 min followed by two washing steps. Blocking with blocking solution (5% foetal bovine serum, 2% bovine serum albumin, 0.1% Triton X-100 and 22.52 mg/ml glycine) was conducted in a wet chamber as well as treatment with the first monoclonal mouse antibody binding to histone H2AX phosphorylated at serine 139 (Abcam 26350) diluted 1:500 in blocking solution at 4 °C overnight. After three washing steps, a second monoclonal goat anti-mouse-IgG antibody coupled with TRITC (Abcam 6786) was used as 1:1000 dilution in blocking solution for 2 h at room temperature in a wet, dark chamber. After two washing steps, slides were mounted (Vectashield) and analysed at 400-time magnification with a Nikon TE2000-E using FITC- and TRITC filter cubes. Main nuclei of micronuclei-free and micronucleated cells as well as micronuclei were evaluated. All values were normalised to main nuclei micronuclei-free control cells, as these cells depict the most normal state with regard to the level of γH2AX-staining.

### Statistics

For statistical analysis, Student’s *t* test or Welch’s *t* test (in the absence of homogenous variances) as well as Mann–Whitney-*U*-test were conducted. All statistical analyses were conducted with IBM SPSS Statistics 26. Data are presented as mean ± standard error of independent experiments. Results were considered significant with *p* ≤ 0.05.

## Results

### Dose-finding

To find appropriate doses with sufficient micronucleus induction but with no relevant cytotoxicity, fixed-cell cytokinesis-block micronucleus test was conducted with all genotoxic agents yielding the following parameters: 20, 30 and 40 nM doxorubicin; 50, 100 and 150 µM tBHP; 0.5, 1 and 2 Gy Radiation; 20, 25 and 30 µg/ml MMS and 1, 2 and 3 nM vinblastine (Supplementary Fig. 1a, b, c, d, e). All doses showed no or only slight cytotoxicity, which was considered to be in an acceptable range.

### Fate of micronuclei

**Fig. 1:**
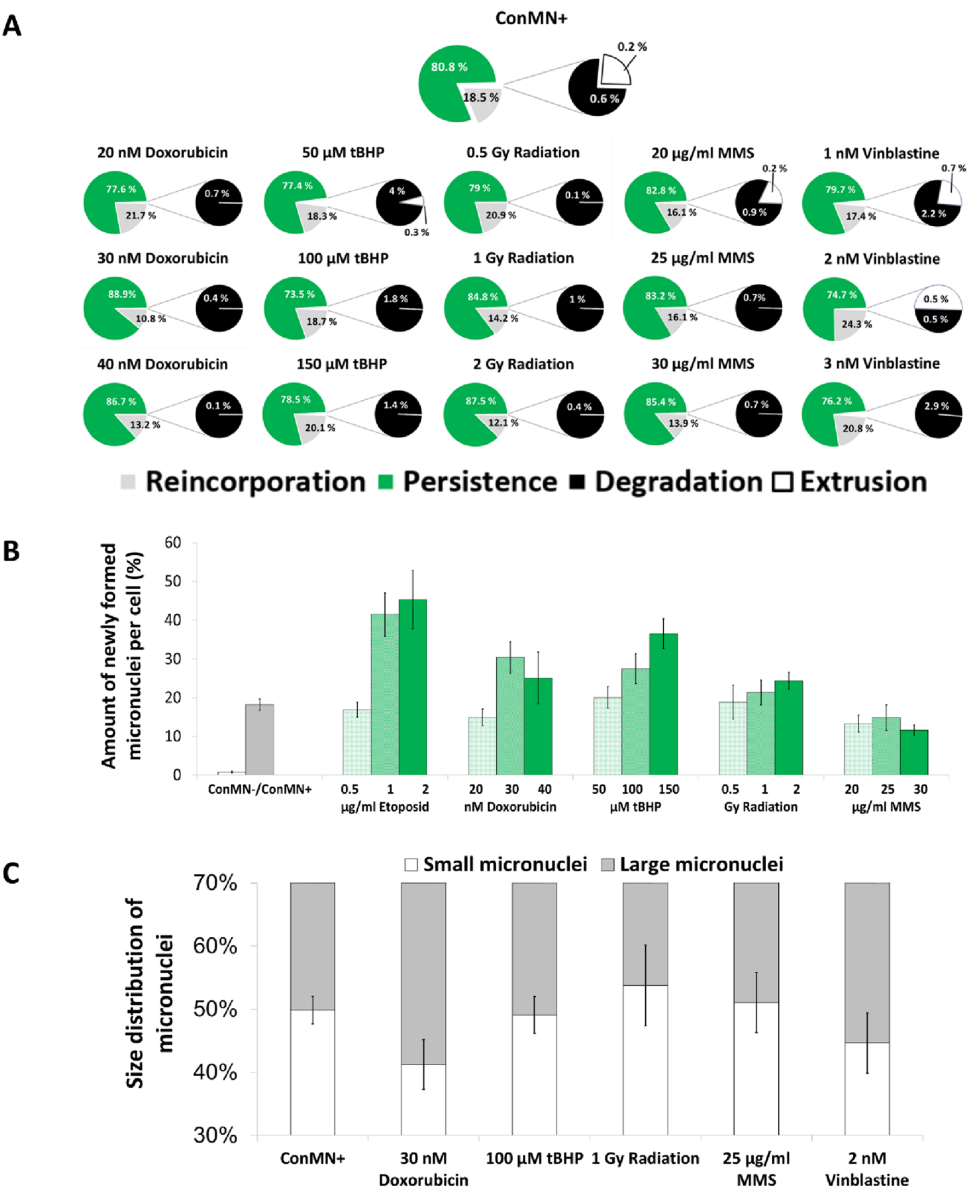
**A** Fates of micronuclei within a cell cycle in HeLa-H2B-GFP cells averaged over all generations based on five experiments: Extrusion, reincorporation, degradation and persistence. **B** Newly formed micronuclei per cell averaged over all generations after administration of doxorubicin, tBHP, radiation, MMS and vinblastine in HeLa-H2B-GFP cells. Number of micronuclei per mitosis in each generation. All presented values are mean out of five experiments with standard error. **C** Size distribution of micronuclei over all generations in ConMN + and after administration of doxorubicin, tBHP, radiation, MMS and vinblastine in HeLa-H2B-GFP cells. All presented values are mean out of three experiments with standard error

The number of observed micronuclei per cell was always ≥ 1 in F0 as only micronucleated cells were chosen for tracking. In F1, a significant increase was observed after medium and high doxorubicin or high MMS treatment, whereas a significant decrease was observed from F2–F5 for all treatments (Supplementary Fig. 2). The most frequently observed fates of micronuclei within a cell cycle over all generations were persistence with a rate between 74.7 and 88.9% of all micronuclei and reincorporation ranging from 10.8 to 24.3% (Fig. 1a). Degradation was only rarely observed, i.e. in 0.1–4% of all cases. The same is true for extrusion, which was only observed in some cases in micronucleated control cells (ConMN +) as well as after treatment with 50 µM tBHP, 20 µg/ml MMS and after 1 and 2 nM vinblastine (Fig. 1a). A clear dose-dependency was not detectable. Only with increasing doses of doxorubicin and radiation, an increase of persistence associated with a decrease of reincorporation was observed, but for MMS, tBHP and vinblastine no or only weak effects could be seen. Furthermore, a decrease of degradation rate was only visible with increasing doses of doxorubicin and tBHP.

New formation of micronuclei after one of the following mitosis was almost never seen in micronuclei-free control cells (ConMN-, i.e. almost no spontaneous micronucleus formation occurred), whereas a clear, significant increase in all micronucleated cells was detectable (Fig. 1b). The number of newly formed micronuclei increased dose-dependently and reached almost 0.5 of all cells with newly formed micronuclei after high doses of doxorubicin. The only exception was treatment with vinblastine, after which only small amount of new micronuclei formed, even less than in ConMN + . In all other cases, the rate of the new micronucleus formation in low treatment groups was similar to ConMN + ranging from 0.15 to 0.25. Micronucleus size was almost equally distributed in ConMN + , approximately the same amount of large and small micronuclei were found (Fig. 1c). This was similar with treatments with medium doses of tBHP and MMS, whereas after radiation 53.8% of all micronuclei were evaluated as small, which was the highest observed value. Doxorubicin treatment caused the highest proportion of large micronuclei with 58.8% of all counted micronuclei followed by vinblastine treatment that showed a rate of 55.3% large micronuclei. Taken together, these results indicate that micronuclei either persist or are being reincorporated into the main nucleus during mitosis, typically they are not degraded or expelled from the cell.

### Fate of micronucleated cells

With the observation time of 96 h, 4–5 cell cycles are expected in normally dividing HeLa-H2B-GFP cells. This was confirmed in ConMN-, in which almost a doubling in cell number for most of the cells was observed for each generation from F0–F4 (Supplementary Fig. 3). For all micronucleated cells, doubling in cell number could be only seen from F0–F1, subsequent generations were only reached by a fraction of the cells, i.e. cell proliferation was decreased (Supplementary Fig. 3).

The rate of micronucleated cells declined from F0–F5 regardless of treatment until almost 0% were reached (Supplementary Fig. 4). The figure also shows the theoretically expected rate of micronucleated cells under the assumption, that all micronucleated cells divide once and form one micronucleated and one non-micronucleated daughter cell (red line). As only micronucleated cells were chosen for analysis in F0, the rate is always 100%. For ConMN + , the rate of micronucleated cells lies below this expected rate in F1, but approaches it again for F2–F5 (Supplementary Fig. 4). After treatment with low doses of doxorubicin, observed and expected rates were very close to each other, while medium and high doses caused more micronucleated cells (Supplementary Fig. 4b). tBHP treatment with low and medium doses caused micronucleated cell values close to the expected ones except for F1, in which they were lower than expected, while high doses of tBHP lead to more micronucleated cells than expected in F2 mainly (Supplementary Fig. 4c). Regardless of dosing, observed rates of micronucleated cells after radiation and MMS treatment were slightly higher than expected in F2 and later (Supplementary Fig. 4d, e). In contrast, after vinblastine treatment less micronucleated cells were observed than expected in F1, while both values approached the expected rates in the subsequent generations (Supplementary Fig. 4f).

**Fig. 2:**
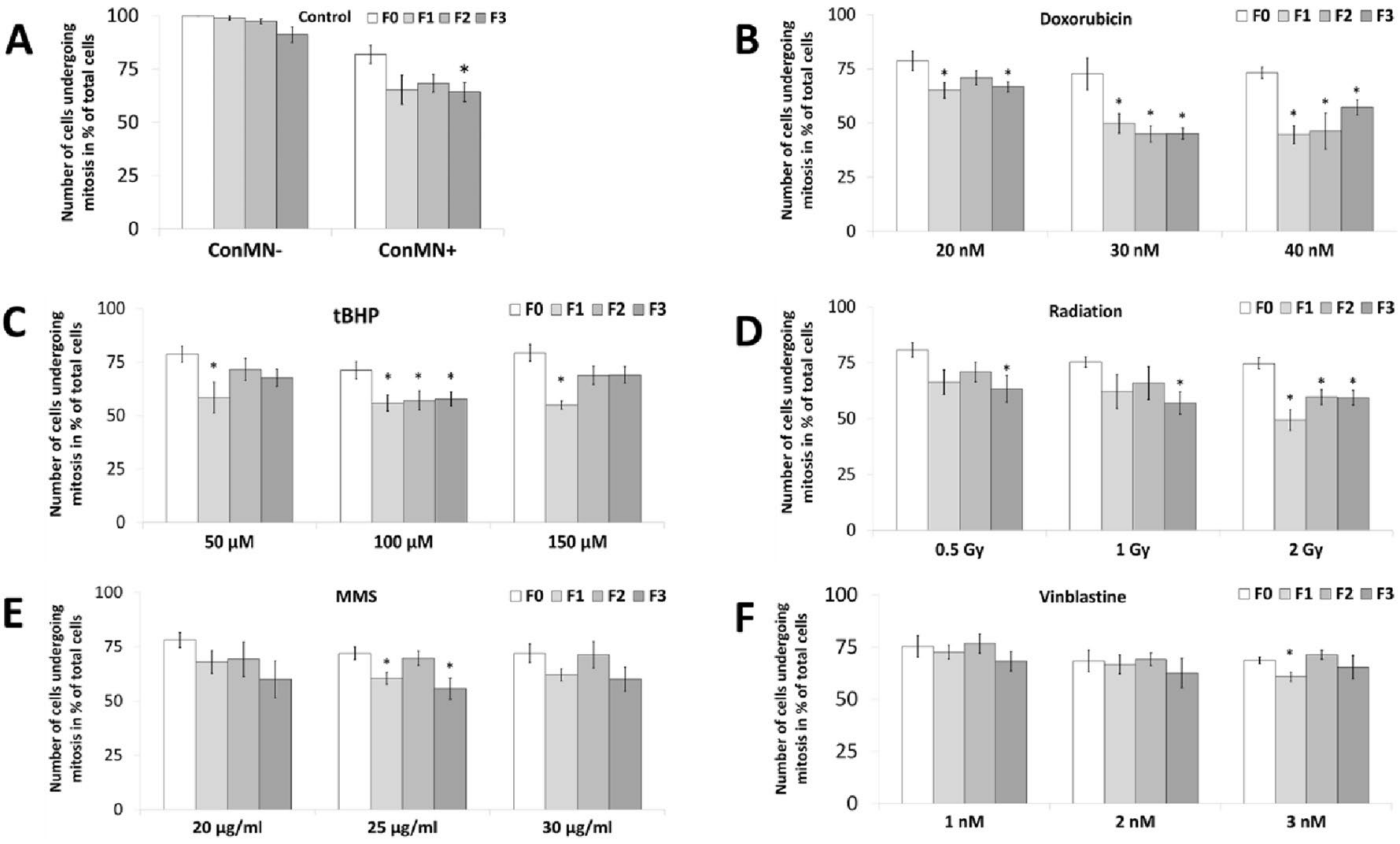
Number of cells undergoing mitosis without any mitotic errors within a generation in % of total cells after administration of **A** doxorubicin, **B** tBHP, **C** radiation, **D** MMS and **E** vinblastine in HeLa-H2B-GFP cells in generation F0-F3. All presented values are mean out of five experiments with standard error. Asterisks represent *p* < 0.05 vs. values in F0 (*t* test)

Number of cells within a generation undergoing mitosis was examined in F0-F3 and was close to 100% in ConMN- in all generations, although a slight decrease was visible in later generations (Fig. 2a). F4 and F5 were excluded for this and other examinations, as events like mitosis could not be detected in some cases due to the end of observation period. In micronucleated cells, mitosis frequency was approximately 80% in F0, but declined to 50–70% within F1–F3, e.g., after doxorubicin treatment (Fig. 2a, b). Interestingly, no further decrease was observed from F2–F3 indicating a potential stabilisation of mitosis frequency. After low and high doses of tBHP an increase in F2 and F3 to ~ 65% was observed after a significant decrease from F0–F1 (Fig. 2c). A similar trend was seen after high dose radiation, for which an increase to 60% in F2 and F3 occurred after a minimum in F1 (Fig. 2d). Only a small decrease from F0–F1 was found after MMS treatment followed by an increase up to almost similar mitosis frequencies than F0 in F2–F3 (Fig. 2e), while mitosis frequencies remained more or less constant over all generations after vinblastine treatments (Fig. 2f).

**Fig. 3:**
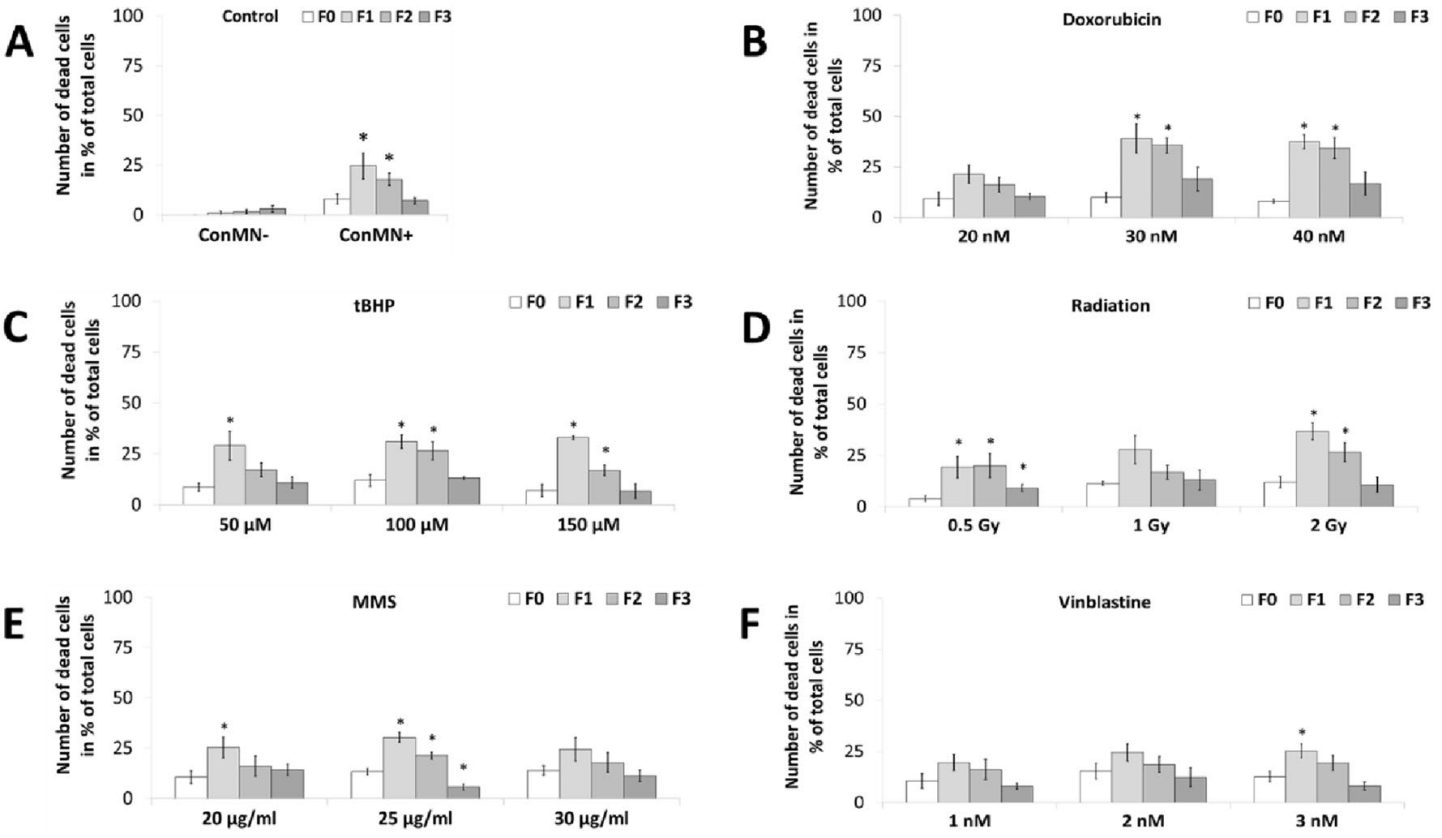
Number of dead cells in each generation in % of total cells after administration of **A** doxorubicin, **B** tBHP, **C** radiation, **D** MMS and **E** vinblastine in HeLa-H2B-GFP cells in generation F0-F3. All presented values are mean out of five experiments with standard error. Asterisks represent *p* < 0.05 vs. values in F0 (*t* test)

Cell death rate in ConMN- was low and increased slowly to almost 5% from F0–F3 (Fig. 3a). For ConMN + , cell death was increased from 5% in F0 to a maximum of 25% in F1 to decrease again to 5% until F3, which is representative for micronucleated cells after most treatments (Fig. 3). However, after medium and high dose doxorubicin treatment, cell death rate reached a peak of 35% in F1, which decreased to 20–25% in F3 (Fig. 3b). After the highest dose of tBHP, a maximum of 30% was reached in F1, while a strong decline to a cell death rate of 5% was seen up to F3 (Fig. 3c). High dose radiation caused a strong increase of cell death up to 35% followed by a strong decline but after low dose radiation in contrast, no difference was seen from F1–F2 (Fig. 3d). After MMS- and vinblastine treatments, no clear difference to ConMN + could be found, although after medium and high doses comparatively high cell death rates were detectable in F0 (Fig. 3e, f).

**Fig. 4:**
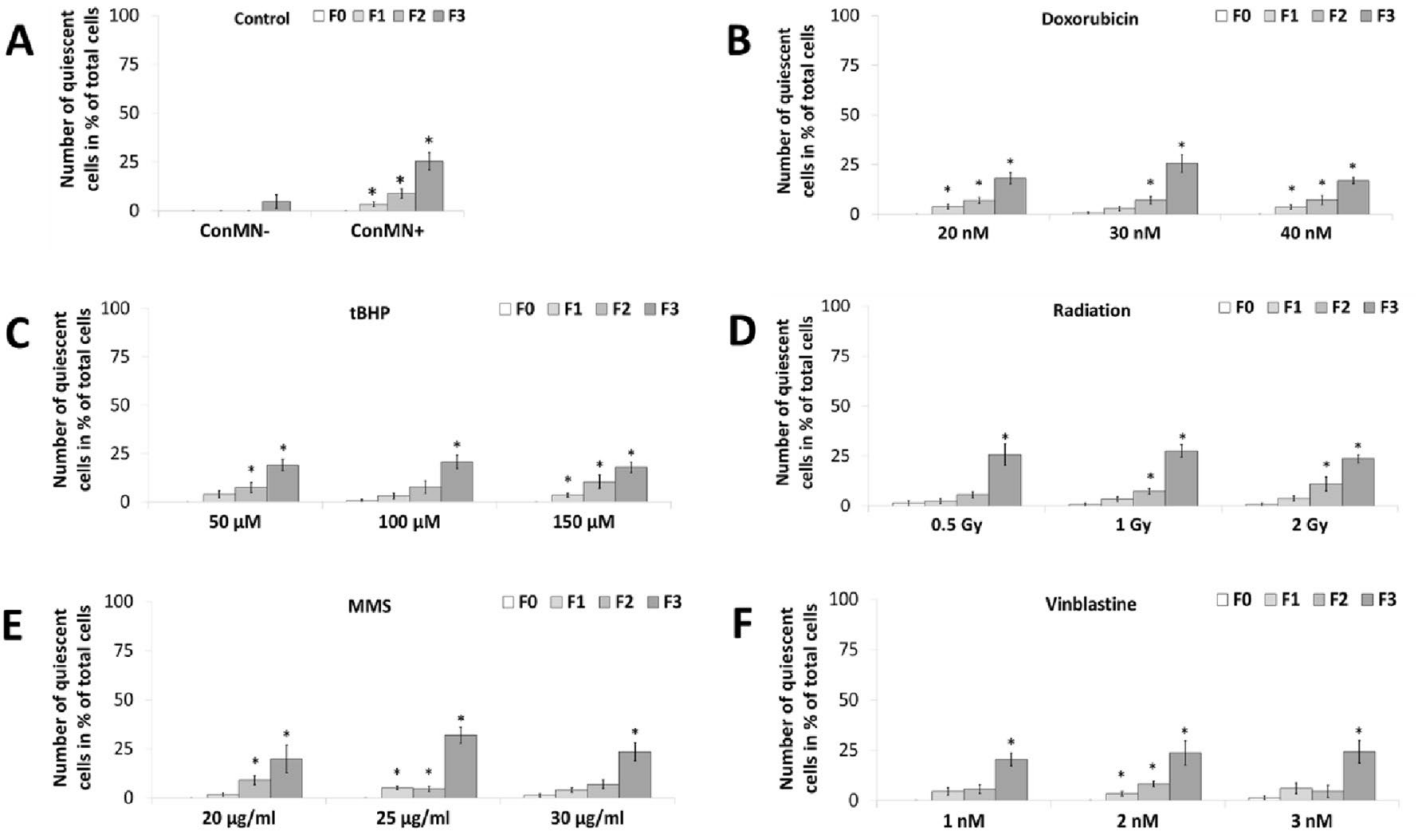
Number of quiescent cells in each generation in % of total cells after administration of **A** doxorubicin, **B** tBHP, **C** radiation, **D** MMS and **E** vinblastine in HeLa-H2B-GFP cells in generation F0–F3. All presented values are mean out of five experiments with standard error. Asterisks represent *p* < 0.05 vs. values in F0 (*t* test)

Number of quiescent cells, i.e. cells with no events like mitosis or cell death taking place, was increased up to approximately 25% after all treatments from F0–F3 and reached significance, which is not surprising, as the end of observation period is closer in F3 than F0 (Fig. 4). Nevertheless, significant differences can be seen when results are compared to ConMN-, for which in F0-F2 almost no and in F3 quiescence was observed in 5% of all cells (Fig. 4a). Between doses and genotoxic agents, only few differences could be found, primarily in F3: After low and high doses of doxorubicin and after all doses of tBHP, quiescence number was reduced (Fig. 4b, c). This is also the case after treatment with low doses of MMS and vinblastine (Fig. 4d, e), whereas after treatment with medium MMS doses, quiescence was observed more often. No deviations from this trend were seen after radiation (Fig. 4f).

Duration of mitosis and duration until next mitosis were analysed. Duration until next mitosis in ConMN- was 19 h in F1 but increased and remained at 22 h in F3 (Supplementary Fig. 5a). Micronucleated cells needed more time until mitosis with a maximum after treatment with medium doses of doxorubicin (27.5 h). But after all treatments, this duration declined again and approached values for ConMN- in F3 and F4 with a range from 20 to 22 h until next mitosis (Supplementary Fig. 5). Mitosis in ConMN- lasted 1.6 h in F0 but increased up to 2.0 h in F3, and remained constant in F4 (Supplementary Fig. 6a). Although all micronucleated cell groups needed 2.0–2.5 h in F0 for mitosis, the duration decreased up to F3 or F4 latest and approached the duration of ConMN- of around 2 h (Supplementary Fig. 6).

### Mitotic errors in micronucleated cells

**Fig. 5:**
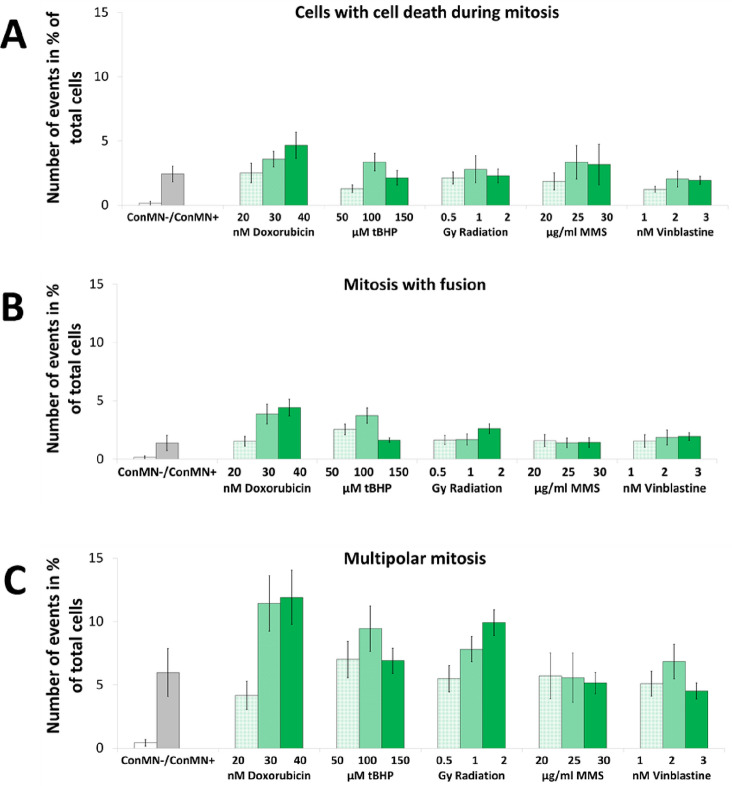
**A** Number of mitotic cells with cell death during mitosis averaged over all generations in % of total cells. **B** Number of mitosis after fusion of two cells averaged over all generations in % of total cells. **C** Number of multipolar mitosis averaged over all generations in % of total cells. All presented values are summarised over all generations after administration of doxorubicin, tBHP, radiation, MMS and vinblastine in HeLa-H2B-GFP cells and are mean out of five experiments with standard error

In addition to the previously described events, also mitotic errors like cell death during mitosis, mitosis after fusion of two or more nuclei during mitosis, or multipolar mitosis occurred. All these abnormal events occurred only very rarely in ConMN- but were often significantly increased in micronucleated cells (Fig. 5a). Cell death during mitosis occurred in 2.4% of all cells in ConMN + , while a dose-dependent increase after treatment with doxorubicin to up to 4.7% of all cells was observed (Fig. 5a). With rates of 3.2 and 2.0%, no clear differences between medium and high dose treatment were observed after treatment with MMS and vinblastine, respectively. After tBHP treatment and radiation, rates for cell death during mitosis reached maximum values at medium doses.

Mitosis after fusion appeared most often after medium and high dose doxorubicin treatment with 4.0–4.5% of total cells, while this event occurred in 1.4% of all cells in ConMN + (Fig. 5b). No clear differences between values of ConMN + and after treatment with MMS or vinblastine were detectable. After high dose radiation mitosis after fusion appeared in 2.6% of all cells. Mitosis after fusion after tBHP treatment showed a maximum at medium dose, as high dose treatment surprisingly only caused a small amount of 1.6% of all cells undergoing this abnormal event.

Multipolar mitosis occurred in almost 6% of all cells in ConMN + (Fig. 5c). Highest values were reached after treatment with medium and high doses of doxorubicin with 11.5%, while after tBHP and vinblastine treatment a maximum could be seen after medium doses at 9.4 and 6.8% of all cells respectively. Only after radiation, a clear dose-dependency was observed up to a maximum after high doses at 9.9%. In contrast, no dose-differences were observed after MMS treatment, for which multipolar mitosis appeared always in around 5.5% of all cells.

### Evidence of degradation in micronuclei

**Fig. 6:**
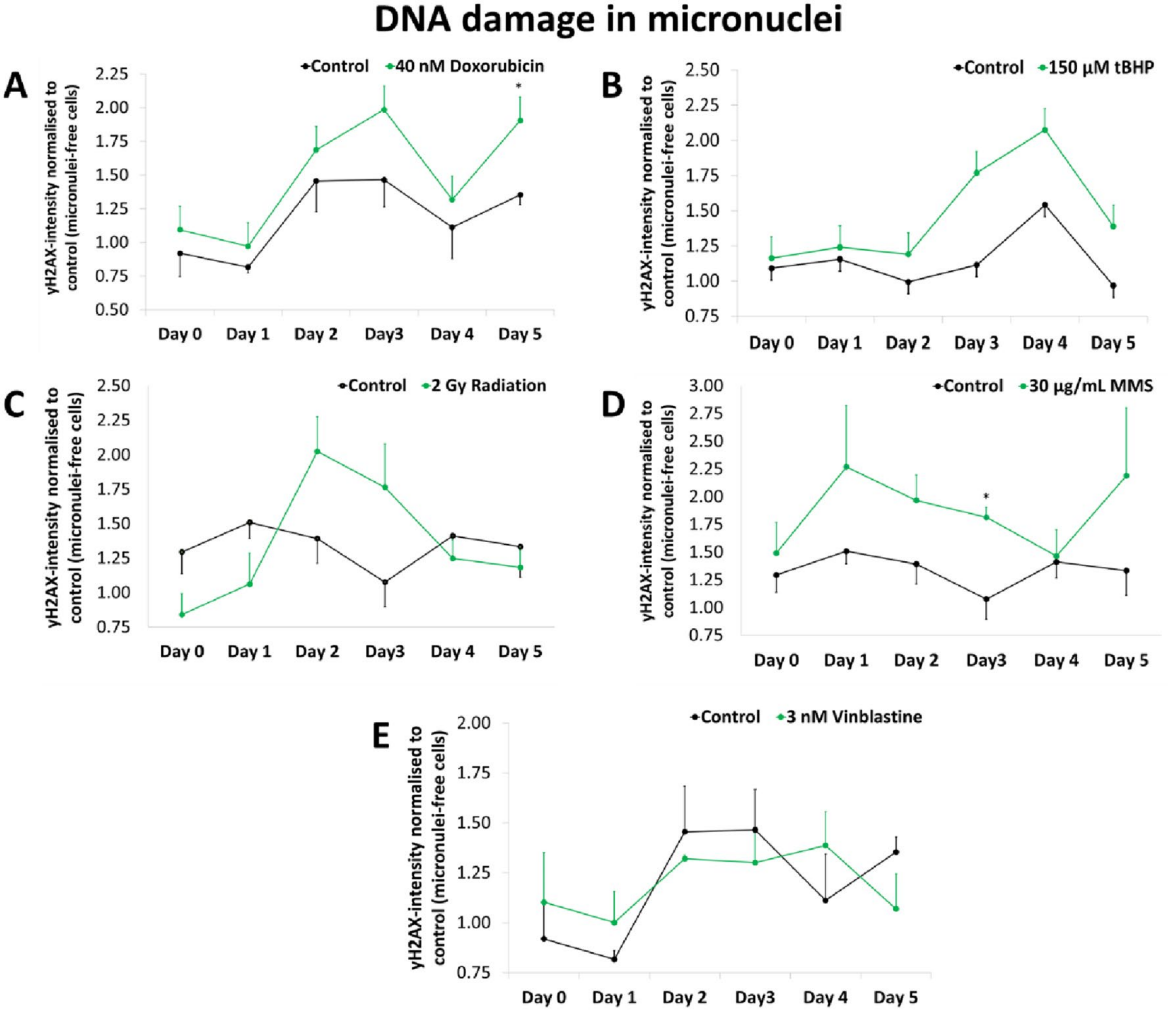
γH2AX-intensity normalised to main nuclei of micronuclei-free control cells in micronuclei of HeLa-H2B-GFP-cells treated with **A** 40 nM doxorubicin, **B** 150 µM tBHP, **C** 2 Gy radiation, **D** 30 µg/ml MMS and **E** 3 nM vinblastine. Green line indicate genotoxic agents, black line indicate respective solvent control cells (DMSO for doxorubicin and MMS experiments, water for tBHP, radiation and vinblastine experiments). All presented values are mean out of three experiments with standard error. Asterisks represent *p* < 0.05 vs. control cells (*t* test)

To further investigate degradation of micronuclei, γH2AX immunostaining was conducted every 24 h for five days after treatment with high doses of the same genotoxic agents as before. After doxorubicin treatment, γH2AX staining increased until day 5 (with an exception on day 4), the intensity was doubled compared to the reference (main nuclei of micronuclei-free control cells). A similar trend but with less DNA damage was observed in micronuclei of control cells (Fig. 6a). Treatment with tBHP caused a doubling of staining intensity in micronuclei until day 4 but levels decreased again on day 5. Again, a similar trend was observed in control micronuclei, although the intensity was lower (Fig. 6b). After radiation, an intensity maximum was reached on day 2 followed by a decrease to day 5, whereby control micronuclei showed only slightly elevated increases in staining intensity with no clear maximum (Fig. 6c). MMS treatment resulted in strong staining increases in micronuclei with a maximum on day 2 with a more than two times higher intensity than the reference followed by a decreasing intensity until day 4, which was elevated again on day 5 (Fig. 6d). Only weak increases of staining intensity could be observed in micronuclei after vinblastine treatment, which was similar to control micronuclei (Fig. 6e). In contrast to micronuclei, only slight differences were observed in micronuclei-free cells compared to reference (which are micronuclei-free control cells). Only immediately after treatment, tBHP caused a strong increase in staining intensity, which was strongly reduced within the next two days (Supplementary Fig. 7). In micronucleated cells, no clear difference to micronucleated control could be observed after all genotoxic agents. Furthermore, the increase in staining intensity compared to the reference value 1 is low (Supplementary Fig. 8).

**Fig. 7:**
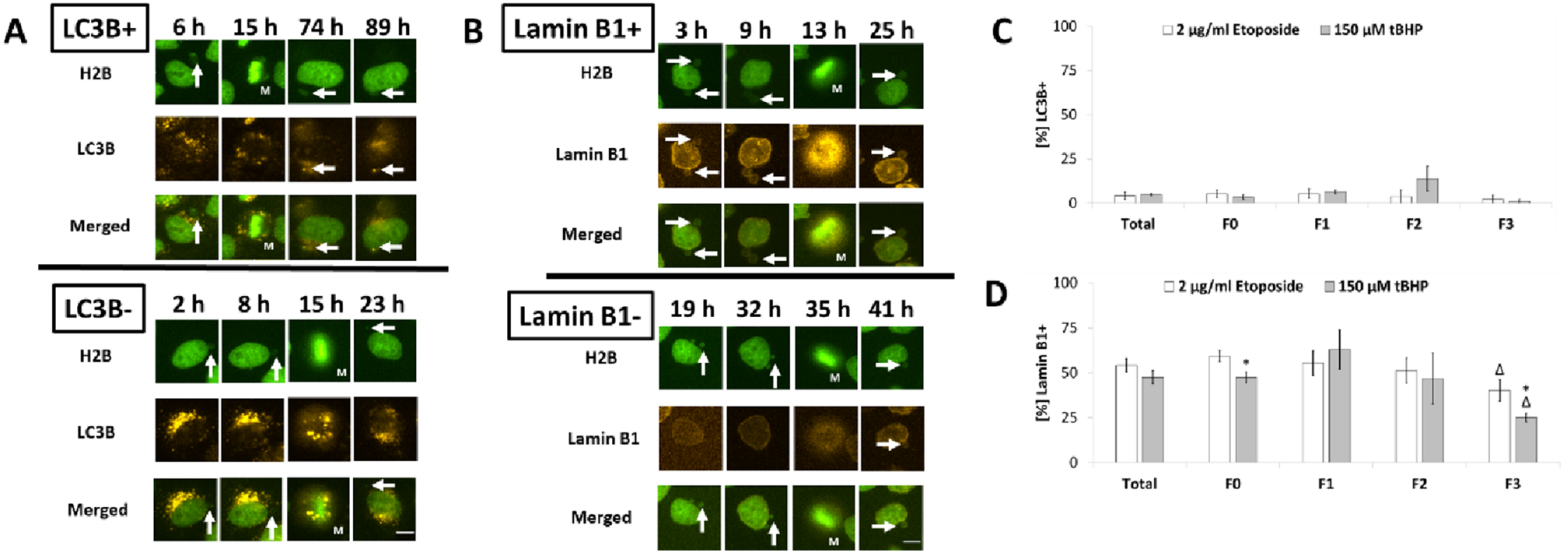
Marker of degradation in micronuclei. **A** HeLa-H2B-GFP cells transduced with reporter gene LC3B-dsRed. **B** HeLa-H2B-GFP cells transduced with reporter gene Lamin B1-dsRed. White arrows indicate micronuclei, M indicate mitosis, scale = 10 µm. **C** Frequency of LC3B-positive micronuclei after administration of etoposide and tBHP. **D** Frequency of Lamin B1-positive micronuclei after administration of etoposide and tBHP. All presented values are mean out of three experiments with standard error. Asterisks represent *p* < 0.05 vs. administration of etoposide; delta represent *p* < 0.05 vs. values in F0 (Mann–Whitney-*U*-Test)

HeLa-H2B-GFP cells transduced with the autophagy marker LC3B or micronuclear integrity marker lamin B1 were used to clarify the role of degradation in micronuclei after two exemplary treatment regimes (Fig. 7a/b). However, co-localisation of LC3B and micronuclei was only rarely observed, independently of treatment or generation (Fig. 7c). Lamin B1 has been demonstrated before as a suitable marker for the integrity of the membrane of main and micronucleus (Hatch et al. 2013). Lamin B1 staining of micronuclei was identified in approximately 50% of all micronuclei, with a decrease from F0 to F3. Treatment seemed to affect the outcome only slightly, but significant less Lamin B1-positive micronuclei were observed after tBHP treatment in F0 and F3 (Fig. 7d).

### Induction of micronucleus extrusion

**Fig. 8:**
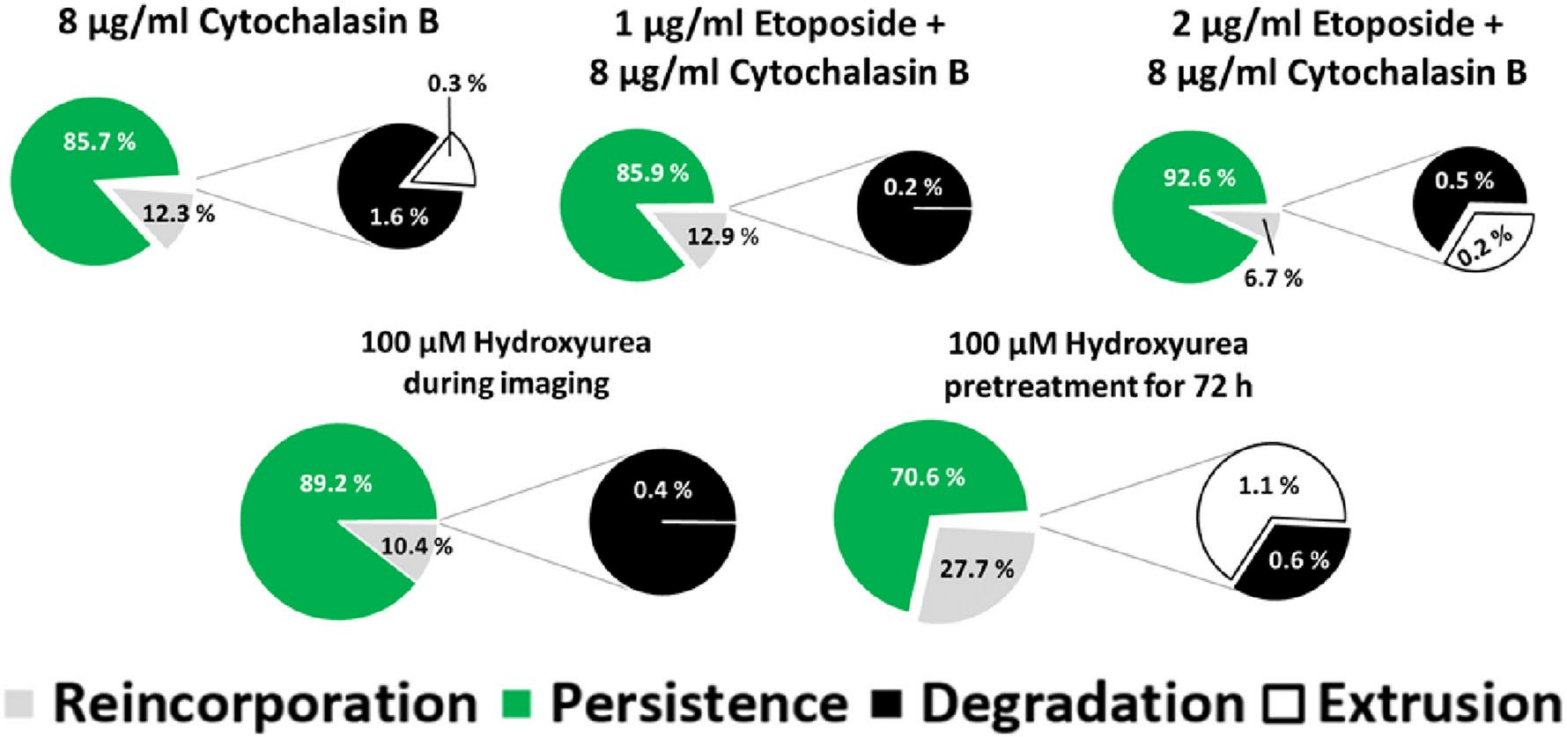
Extrusion of micronuclei. Fates of micronuclei within a cell cycle in HeLa-H2B-GFP after administration of cytochalasin B, etoposide and hydroxyurea averaged over all generations based on three experiments: Extrusion, reincorporation, degradation and persistence

Extrusion of micronuclei was reported in some studies, but only under special conditions, e.g., after treatment with high doses (8 µg/ml) of cytochalasin B in combination with a genotoxic agent or after treatment with low doses (100 µM) doses of hydroxyurea for 72 h before or during the imaging period (Nito et al. 1988; Shimizu et al. 2000). To further elucidate on which parameters these effects depend, we investigated extrusion of micronuclei under these special conditions with our test system. Only after hydroxyurea pre-treatment for 72 h, a slight increase of extrusion of 1.1% was observed (Fig. 8a). After all other treatments, extrusion rates varied from 0.0 to 0.3% and can be considered very low, which was similar to the genotoxic agents tested before (Fig. 1a). Furthermore, degradation was seen on a similar level compared to the treatments investigated before. Interestingly, pre-treatment with 100 µM hydroxyurea for 72 h resulted in the highest observed rate of reincorporation (27.7%). Treatment with 100 µM hydroxyurea during imaging and the combination of 2 µg/ml etoposide and 8 µg/ml cytochalasin B however lead to low rates of reincorporation with 10.4 and 6.7% respectively together with high frequency of persistence with 89.2 and 92.6% respectively.

## Discussion

Classically, the micronucleus frequency has been used as biomarker for genotoxicity of various substances like pharmaceuticals or chemicals (OECD 2016; Reimann et al. 2021; Reimann et al. 2020a, b). Additionally, in the last years, an increasing number of studies have been published revealing more and more details on the biological relevance and the cellular consequences of micronucleus formation (Guo et al. 2022; Krupina et al. 2021; Kwon et al. 2020). However, long-term observations of micronuclei and micronucleated cells are still rare, so a number of questions about the fate of micronuclei are still open. In a previous study, we investigated the fate of micronuclei and micronucleated cells in HeLa-H2B-GFP-cells after treatment with etoposide (Reimann et al. 2020a, b). In the present study, we aimed at investigating whether these findings are substance-specific or can be generalized by using various different genotoxic agents. Furthermore, degradation and extrusion of micronuclei were investigated mechanistically. The genotoxic agents applied in the present study cause DNA damage via different mechanisms, therefore potential differences in micronuclear fate due to the origin of DNA damage could be addressed.

Mitosis after micronucleus induction was observed in previous studies up to the second mitosis, while in this study the ability for cell division was shown up to four mitoses after administration of all genotoxic agents (Crasta et al. 2012; Soto et al. 2018; Thompson and Compton 2011). However, a reduction of mitosis rate was observed, which remained constant from the second to fourth mitosis. Furthermore, mitosis (and interphase) duration was increased in micronucleated cells but decreased again with further cell cycles, which is in line with other studies in HeLa or nasopharyngeal carcinoma cells (Huang et al. 2011; Utani et al. 2010). There is evidence that kinetochore-positive micronuclei with whole chromosomes are more prone to cell cycle delay than kinetochore-negative micronuclei with chromosomal fragments (Jiang et al. 2019). However, no changes in mitosis rate and duration was observed in mouse embryo cells indicating a strong dependence on cell line and differentiation state (Vázquez-Diez et al. 2016). Cell death rate was increased in micronucleated cells, whereby the aneugen vinblastine showed the weakest response and the clastogen doxorubicin the strongest. Also other studies showed evidence that there is substance-dependency on cell death rate (Yasui et al. 2010). Again, kinetochore-positive micronuclei were more affected by cell death than kinetochore-negative micronuclei, while in PTK1 cells with no micronuclear membrane damage only slight changes in cell death rate were observed indicating a dependence of cell line or rupture of micronuclei on cell death (He et al. 2019; Jiang et al. 2019). As similar trends for mitosis and cell death rate were found in micronucleated cells after etoposide treatment, a consistent effect can be assumed in the used HeLa cells (Reimann et al. 2020a, b). As mitosis rate remained constant after the second mitosis and cell death rate in addition stabilised or was even reduced after two cell cycles, it can be expected that micronucleated cells can at least partially survive and proliferate.

After treatment with all genotoxic agents, persistence and reincorporation were the major fates of micronuclei, persistence occurred more often than reincorporation. No clear substance-specific differences were observed, although there may be a decrease in reincorporation rate with increasing doses of etoposide, doxorubicin and radiation. The finding of persistence and reincorporation being the dominant fates is in line with other reports using live cell imaging experiment (Crasta et al. 2012; Soto et al. 2018; Utani et al. 2010). Interestingly, the rates of persistence and reincorporation are highly variable depending on cell type ranging from almost no reincorporation but high frequency of persistence to the opposite (Huang et al. 2012; Vázquez-Diez et al. 2016). Even different variants of the same cell line may show different frequencies for persistence of micronuclei (Huang et al. 2011). Persistence of micronuclei over multiple cell cycles also demonstrate the fact that micronucleus formation impacts the cell over a longer period and that cells with damaged DNA can sustain for longer periods. Furthermore, evidence for senescence was seen in micronucleated cells in this study after administration of genotoxic agents. Recent studies have shown that cGAS could, apart from autophagy, induce senescence of cells via cGAS-STING when sensing cytosolic DNA, which might be an explanation for senescence in micronuclei (Glück et al. 2017; Yang et al. 2017). However, despite of senescence, cell death and the additionally observed mitotic errors, more than 50% of all cells proliferated during observation period of four to five cell cycles.

Degradation of micronuclei was only rarely observed in our experiments, irrespective of the treatment, which is in line with similar experiments using live cell imaging (Crasta et al. 2012; Yasui et al. 2010). However, several studies indicate co-localisation of micronuclei with autophagy marker LC3B or lysosomal marker LAMP2, which was taken as an indication of degradation of micronuclei (Rello-Varona et al. 2012; Sagona et al. 2014). Inhibition of lysosomes was linked with a reduction of DNA damage levels in micronuclei, which was demonstrated in the form of less γH2AX-positive micronuclei. According to the authors, lysosomal enzymes may be involved in the fragmentation of micronuclear DNA after fusion of lysosomes and micronuclei, which was observed as well (Maass et al. 2018). Autophagy of the main nucleus and the micronucleus was furthermore described in yeast as essential step of autophagy (Otto and Thumm 2020). Recent publications revealed that cGAS (cyclic GMP-AMP synthase), a sensor of cytosolic DNA and mediator of immune response, can interact with LC3 and degrade micronuclei by autophagy (Zhao et al. 2021). Sensing DNA via cGAS requires that DNA from micronuclei is accessible for cytosolic proteins. Rupture of micronuclei would allow contact of cytosolic and micronuclear material (Hatch et al. 2013). Our experiments with the micronuclear membrane marker lamin B1 confirmed that integrity of the micronuclear membrane is impaired in many micronuclei (Hatch et al. 2013). An important factor for rupture of micronuclei is the localisation of micronuclei during mitosis, whereby peripheral localisation leads to an decrease of so-called non-core proteins in the micronuclear membrane and to an increase of rupture (Liu et al. 2018). A further important factor is the homeostasis of ESCRTIII, which is necessary for (micro-) nuclear membrane integrity but impaired at micronuclei (Willan et al. 2019). Additional studies are necessary to clarify the discrepancy between findings of co-localisation of micronuclei and autophagy markers (suggesting degradation of the micronuclei) and the lack of observed degradation of micronuclei in several live-imaging studies. Cell lines with reporter genes e. g. cGAS-STING or LAMP1/2 could be interesting future study objects, however, a reporter gene cell line with LC3B in this study failed to find an association with autophagy and micronuclei.

The rupture of micronuclei is associated with impaired replication, transcription and damaged DNA (Luijten et al. 2018; Okamoto et al. 2012; Utani et al. 2007). The massively damaged DNA in ruptured micronuclei is part of the phenomenon called chromothripsis, whereby the damaged micronuclear DNA undergoes error-prone repair (e.g., via non-homologous end-joining) and gets reincorporated during mitosis causing chromosomal instability (Luijten et al. 2018). The frequency of chromothripsis is tumour-dependent and ranges from 0 to 25% in with a mean of 5% (Stephens et al. 2011; Zhang et al. 2013). yH2AX-staining of micronuclei in the present study and other reports revealed that micronuclear DNA damage is repaired only slowly compared to main nuclei (of micronuclei-free as well as micronucleated cells) and can persist for several cell cycles indicating a central feature of chromothripsis, whereby the cells in this and other live-imaging studies furthermore showed the ability to reincorporate (Terradas et al. 2009).

Like degradation, extrusion was seen only rarely or never, regardless of the treatment. In addition to the standard protocol of treatment with different genotoxic agents, extrusion induction was also investigated under two types of special conditions, based on literature reports on high extrusion rates. First, high doses of cytochalasin B were used with and without high doses of etoposide. According to the literature, treatment of cytochalasin B with a genotoxic agent (mitomycin C or vincristine) should lead to the extrusion of the main nucleus and micronuclei (Nito et al. 1988). No extrusion could be observed in HeLa-H2B-GFP cells although the same doses of cytochalasin B were used as mentioned in the literature. One explanation might be that even higher doses of cytochalasin B would have been necessary for the HeLa cells, but this could result in unacceptable cytotoxicity. Extrusion of genetic material could furthermore be dependent on the cell line and its physiological properties, as e. g. extrusion of micronuclei was observed in mouse bone marrow erythrocytes, which physiologically extrude their cell nucleus (Schriever-Schwemmer et al. 1997). A second approach to induce extrusion was treatment with low doses of hydroxyurea for a longer period before or during imaging. While low hydroxyurea doses (< 150 µM) are known to eliminate extrachromosomally amplified DNA, higher doses may increase DNA amplification (Von Hoff et al. 1992). Again, no effect of the treatment could be identified. A reason for this might be the specific extrusion mechanism of hydroxyurea treatment in some cell types: When DNA of double minutes gets damaged by hydroxyurea, aggregates of double minutes were formed via homologous recombination, which can be removed from the cell during extrusion (Oobatake and Shimizu 2019). As possibly no or only few micronuclei in HeLa-H2B-GFP cells comprise double minutes, this mechanism may not be relevant. Taken together, extrusion of micronuclei seems to be only important to special cell lines or at certain types of micronuclei. As extrusion is no important mechanism for the elimination of micronuclei, damaged DNA remains in cells and could promote tumour evolution.

No clear substance-related effects on the fate of micronuclei and micronucleated cells could be observed. After administration of the clastogens etoposide and radiation, the size of micronuclei was smaller compared to treatment with the aneugen vinblastine, which was expected due to the formation of chromosomal fragments after treatment with clastogens. However, treatment with the clastogen doxorubicin caused larger micronuclei, which could possibly be explained by formation of micronuclei containing several chromosomal fragments. The other observed endpoints in this study showed no effect depending on whether the substance was a clastogen or aneugen. A recent study could demonstrate that length of chromosomes and gene density were important contributors of membrane stability. Larger micronuclei showed increased lamin B1 level and nuclear pore density, which are both known factors for an increased probability of ruptured micronuclei (although other factors may be important as well; Mammel et al. 2022). It can be speculated, if clastogens, which cause smaller micronuclei with chromosomal fragments, also leads to increased micronuclei rupture. But as e.g. the clastogen doxorubicin deviated from this trend by inducing larger micronuclei, no clear conclusion can be made.

The results of this study showed that despite of cell death, senescence and fatal mitotic errors occurring in some micronucleated cells, a high number of micronucleated cells formed after administration of a genotoxic agent can survive and produce daughter cells with and without micronuclei. As micronuclear DNA shows a high degree of DNA damage, it can be assumed that even after reincorporation no normal state can be regained, while persistence of micronuclei also leads to a persistence of the enclosed DNA damage. Depending on the transcription/translation activity and the amount of DNA damage in the micronucleus, both persistence and reincorporation might produce stable and normal cells, but it might also lead to genomic instability, a known enabling characteristic of cancer (Hanahan and Weinberg 2011; Ye et al. 2019). No clear substance-dependent effects were identified, as similar trends for the fate of micronuclei and micronucleated cells were observed after administration of all genotoxic agents investigated.

## Supplementary Information

Below is the link to the electronic supplementary material.

Supplementary file1 (DOCX 1918 KB)

## Data Availability

The datasets generated during and/or analysed during the current study are available from the corresponding author on reasonable request.
