# Supplementary material for: Fate of micronuclei and micronucleated cells after treatment of HeLa cells with different genotoxic agents
Source: Arch Toxicol. 2022 Dec 23;97(3):875–89. doi: 10.1007/s00204-022-03433-9 (PMC9968706; doi:10.1007/s00204-022-03433-9)
Supplement: Supplementary file 1 — Supplementary file1 (DOCX 1918 KB) [file 204_2022_3433_MOESM1_ESM.docx]

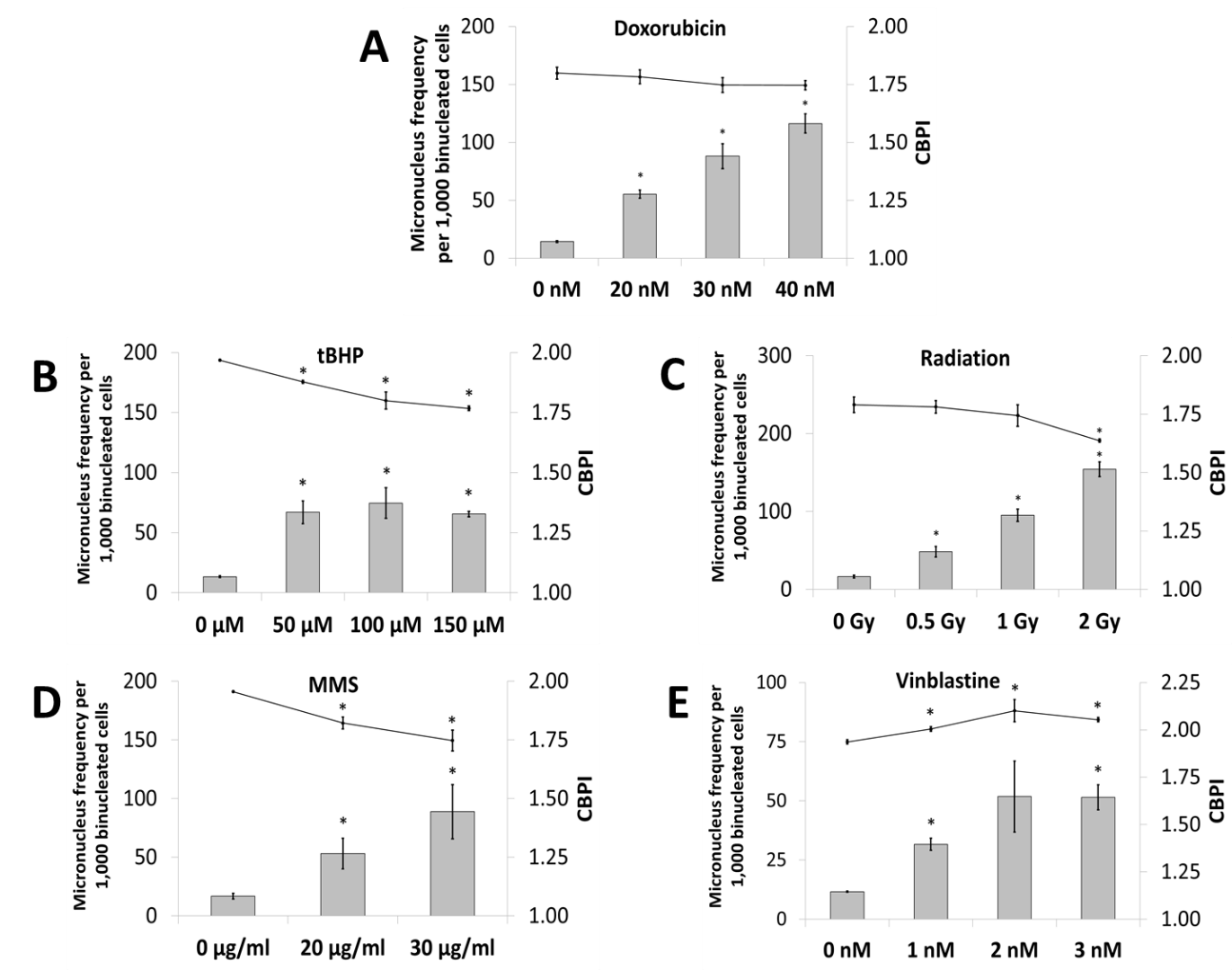


**Supplementary Fig. 1.** Micronucleus frequency (grey bar) per 1,000 binuclear cells and CBPI (black line) after administration of (A) doxorubicin, (B) tBHP, (C) radiation, (D) MMS and (E) vinblastine in HeLa-H2B-GFP cells. Mean of three independent experiments ± standard error. Asterisks represent p<0.05 vs. administration of genotoxic agent (t-test).


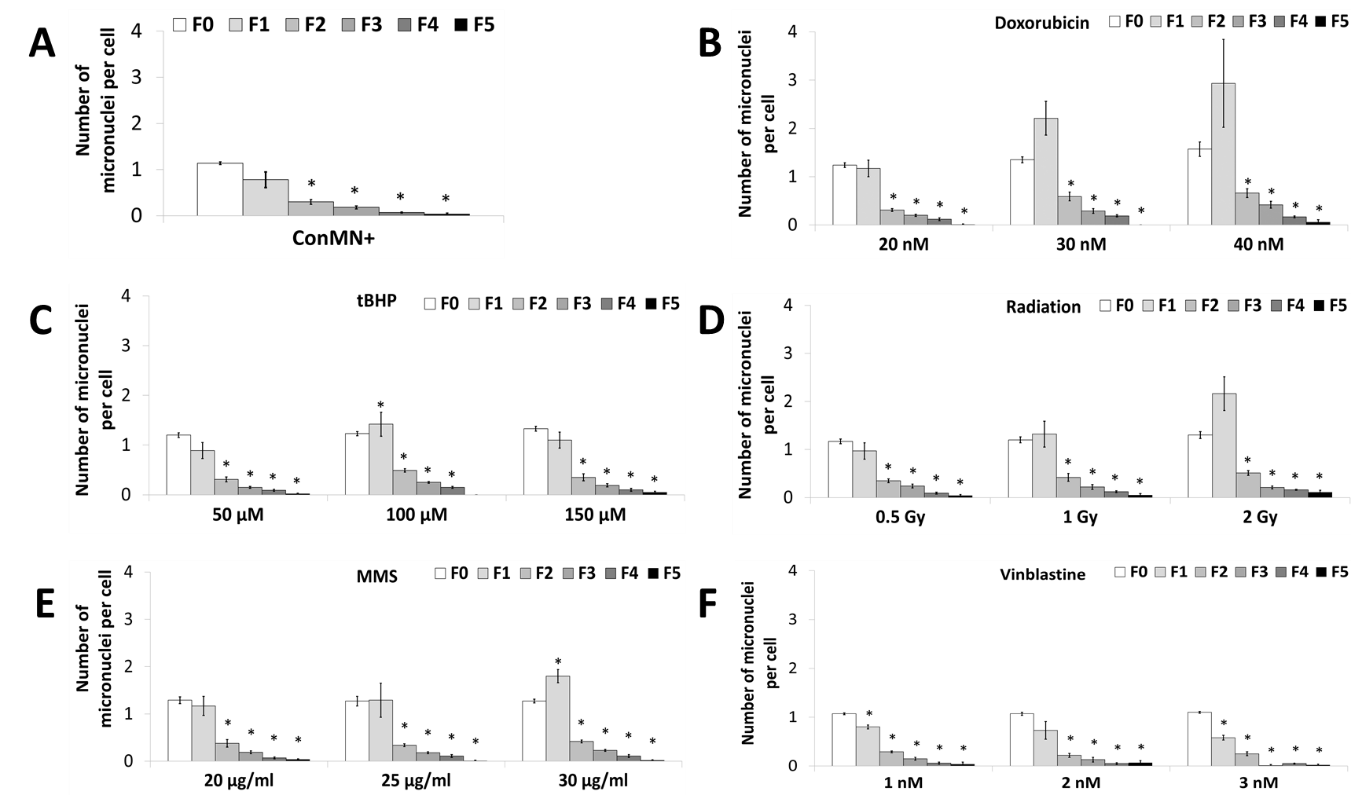


**Supplementary Fig. 2.** Micronuclei per cell in each generation after administration of (A) doxorubicin, (B) tBHP, (C) radiation, (D) MMS and (E) vinblastine in HeLa-H2B-GFP cells. All presented values are means out of five experiments with standard error. Asterisks represent p<0.05 vs. values in F0 (t-test).


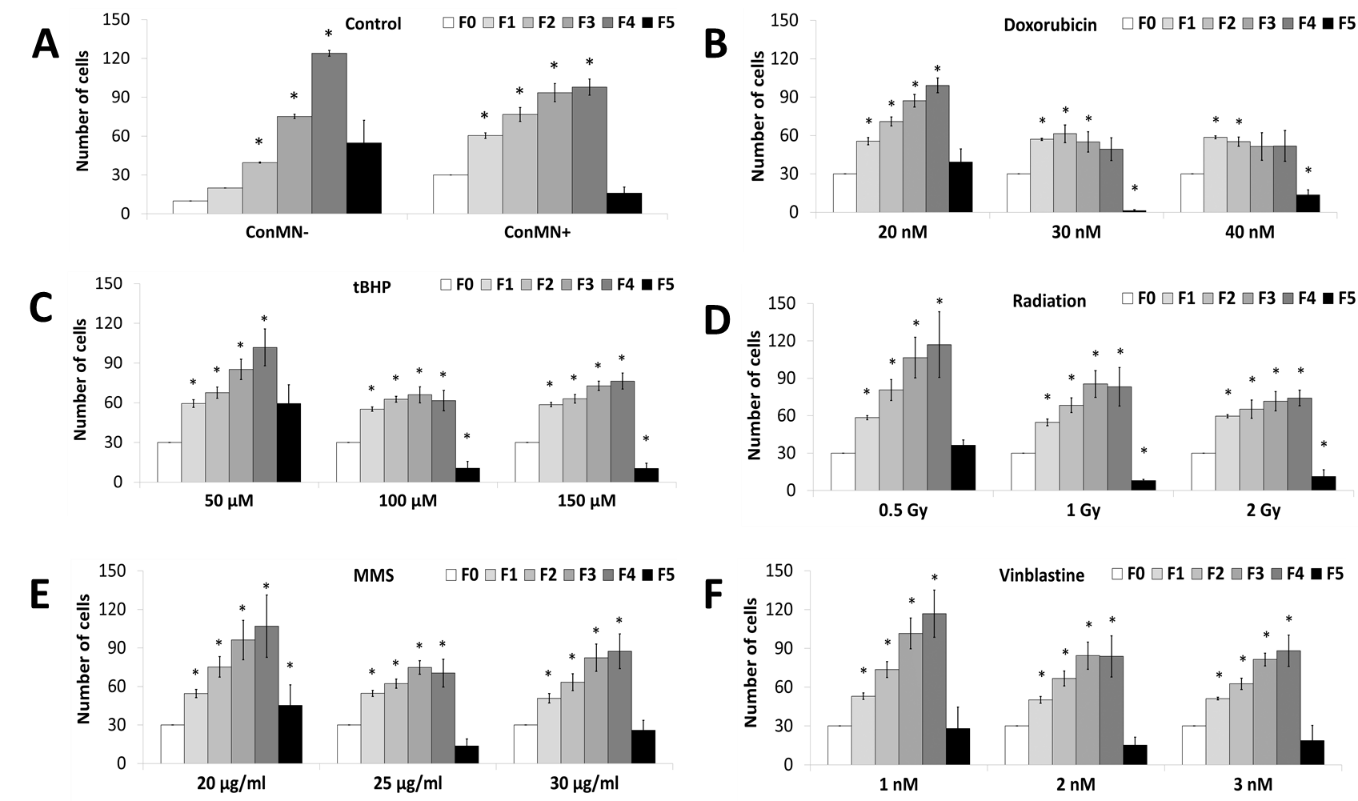


**Supplementary Fig. 3.** Number of cells in each generation after administration of (A) doxorubicin, (B) tBHP, (C) radiation, (D) MMS and (E) vinblastine in HeLa-H2B-GFP cells in generation F0-F5. All presented values are mean out of five experiments with standard error. Asterisk represents p<0.05 vs. values in F0 (t-test).


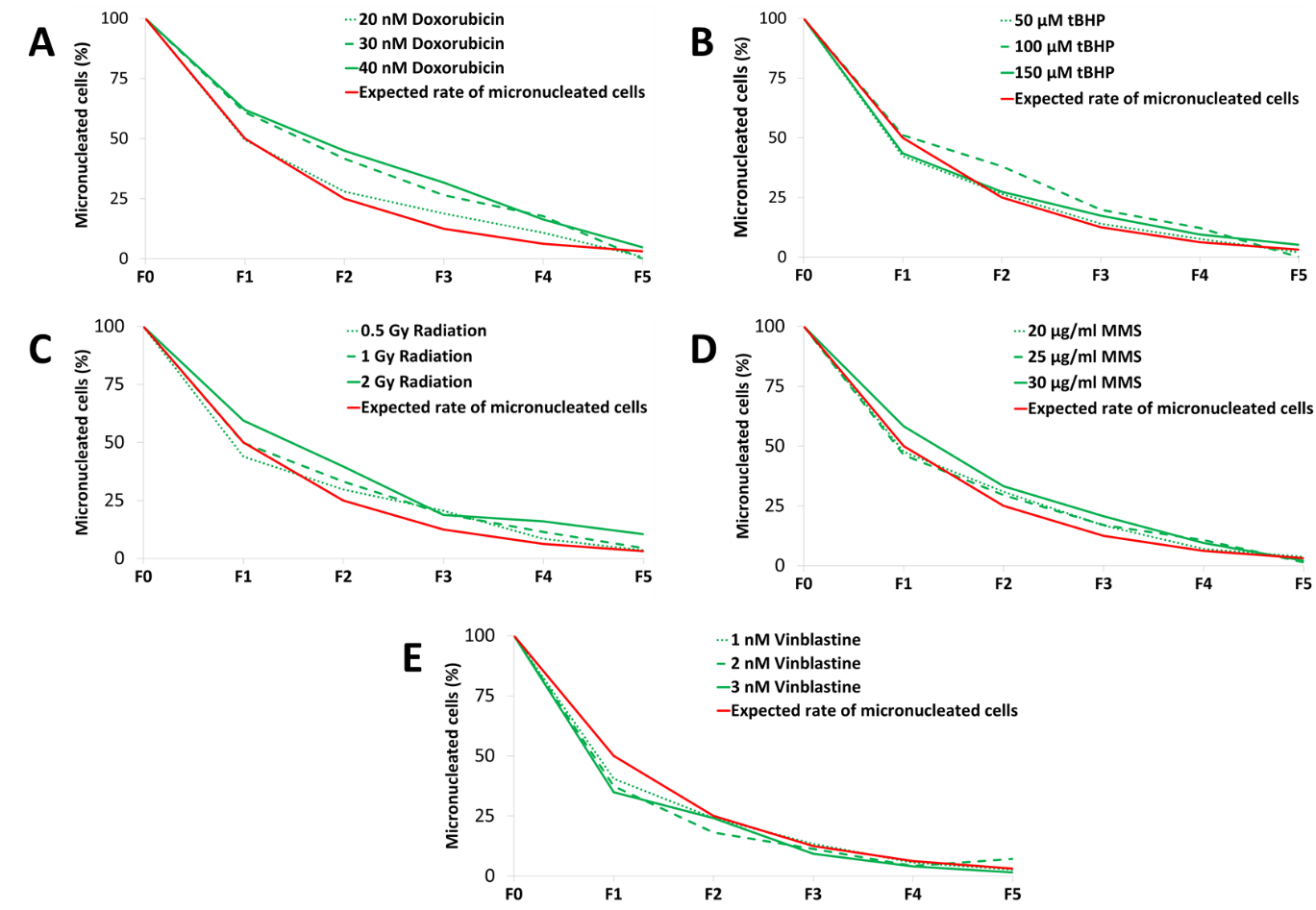


**Supplementary Fig. 4.** Percentage of micronucleated cells after administration of (A) doxorubicin, (B) tBHP, (C) radiation, (D) MMS and (E) vinblastine in HeLa-H2B-GFP cells in generation F0-F5 averaged from five experiments. The red line indicates expected rates of micronucleated cells considering the dilution of micronuclei after each mitosis.


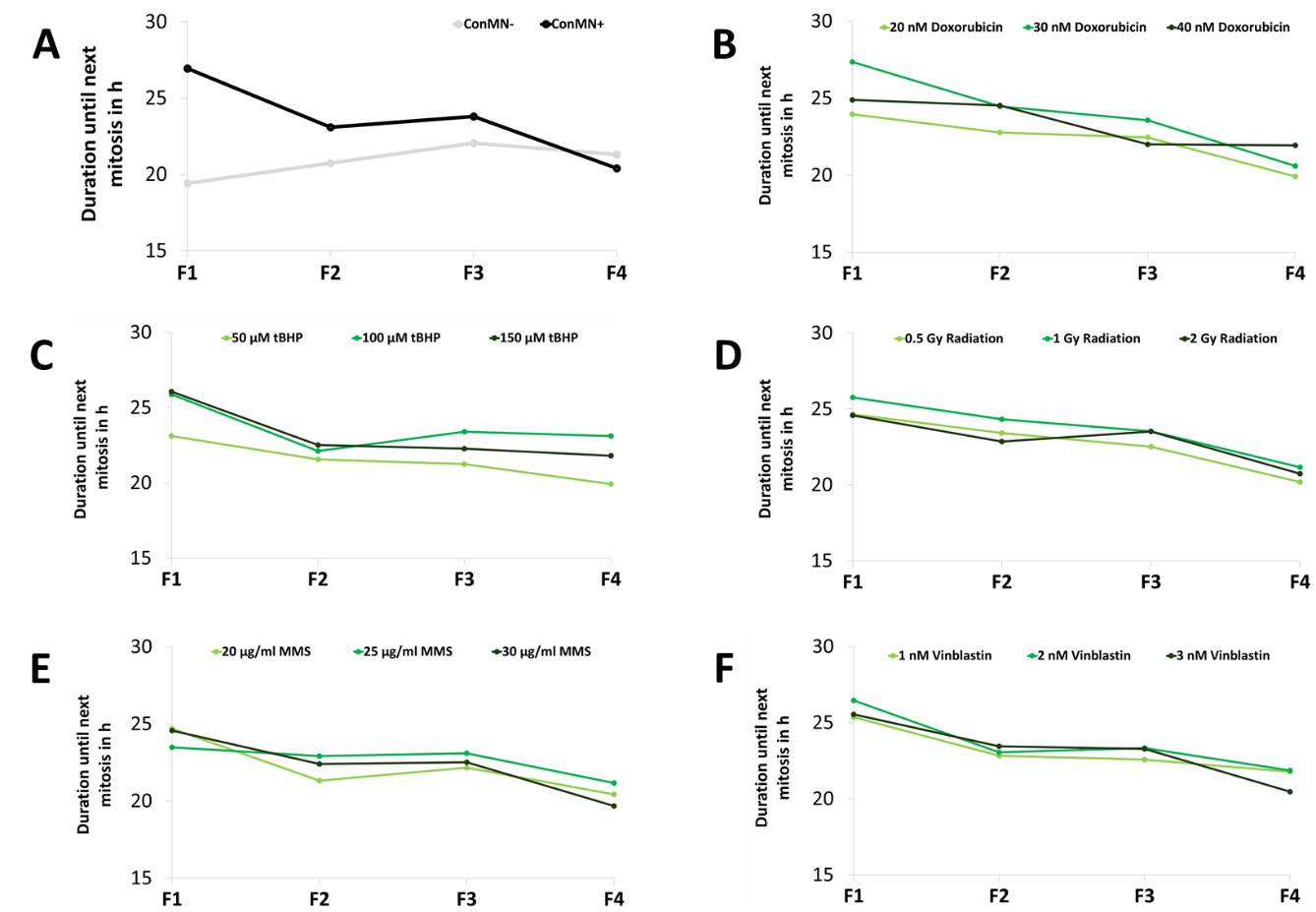


**Supplementary Fig. 5.** Duration until next mitosis in h after administration of (A) doxorubicin, (B) tBHP, (C) radiation, (D) MMS and (E) vinblastine in HeLa-H2B-GFP cells in generation F1-F4 averaged from five experiments.


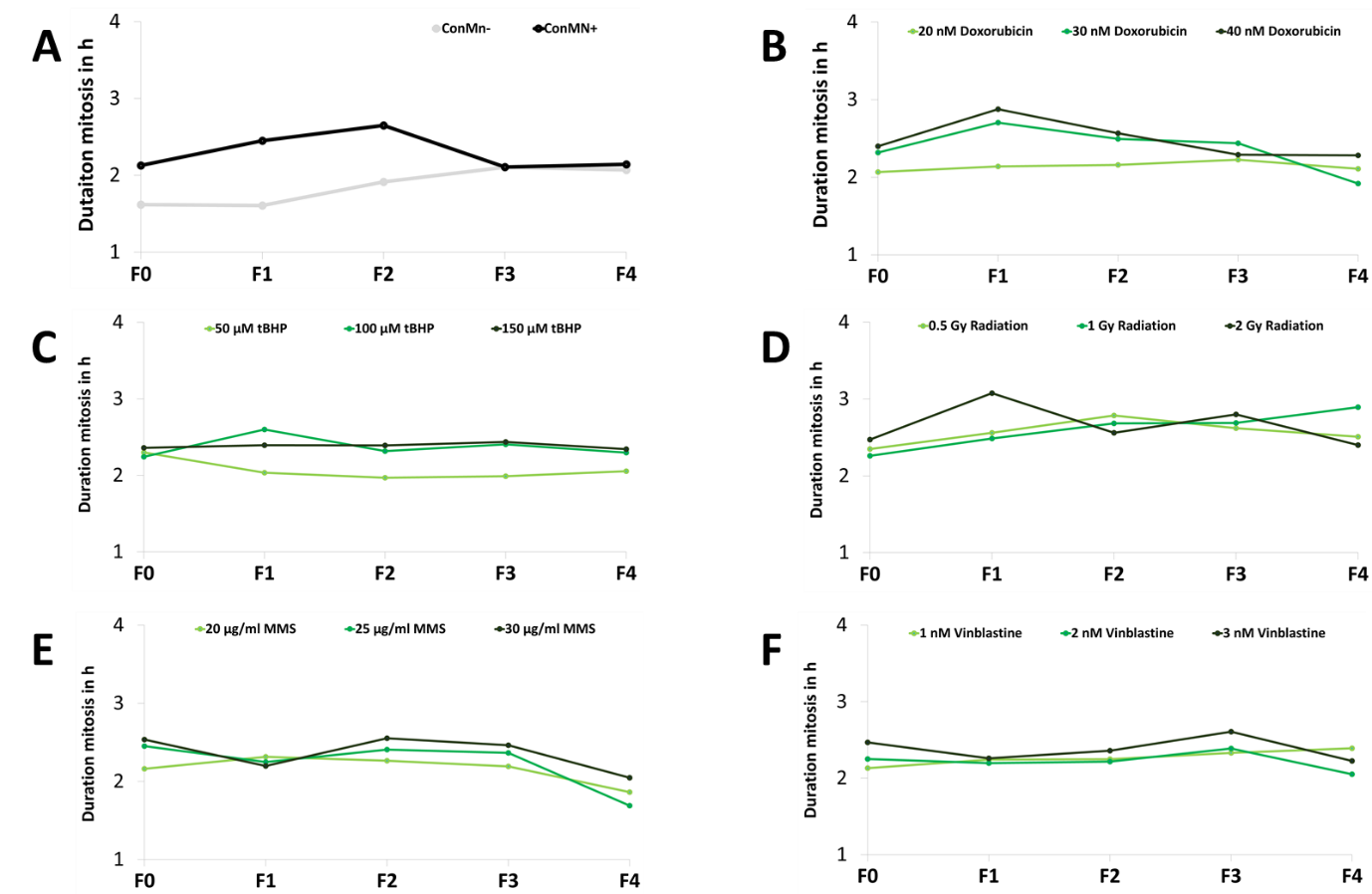


**Supplementary Fig. 6.** Duration of mitosis in h after administration of (A) doxorubicin, (B) tBHP, (C) radiation, (D) MMS and (E) vinblastine in HeLa-H2B-GFP cells in generation F0-F4 averaged from five experiments.


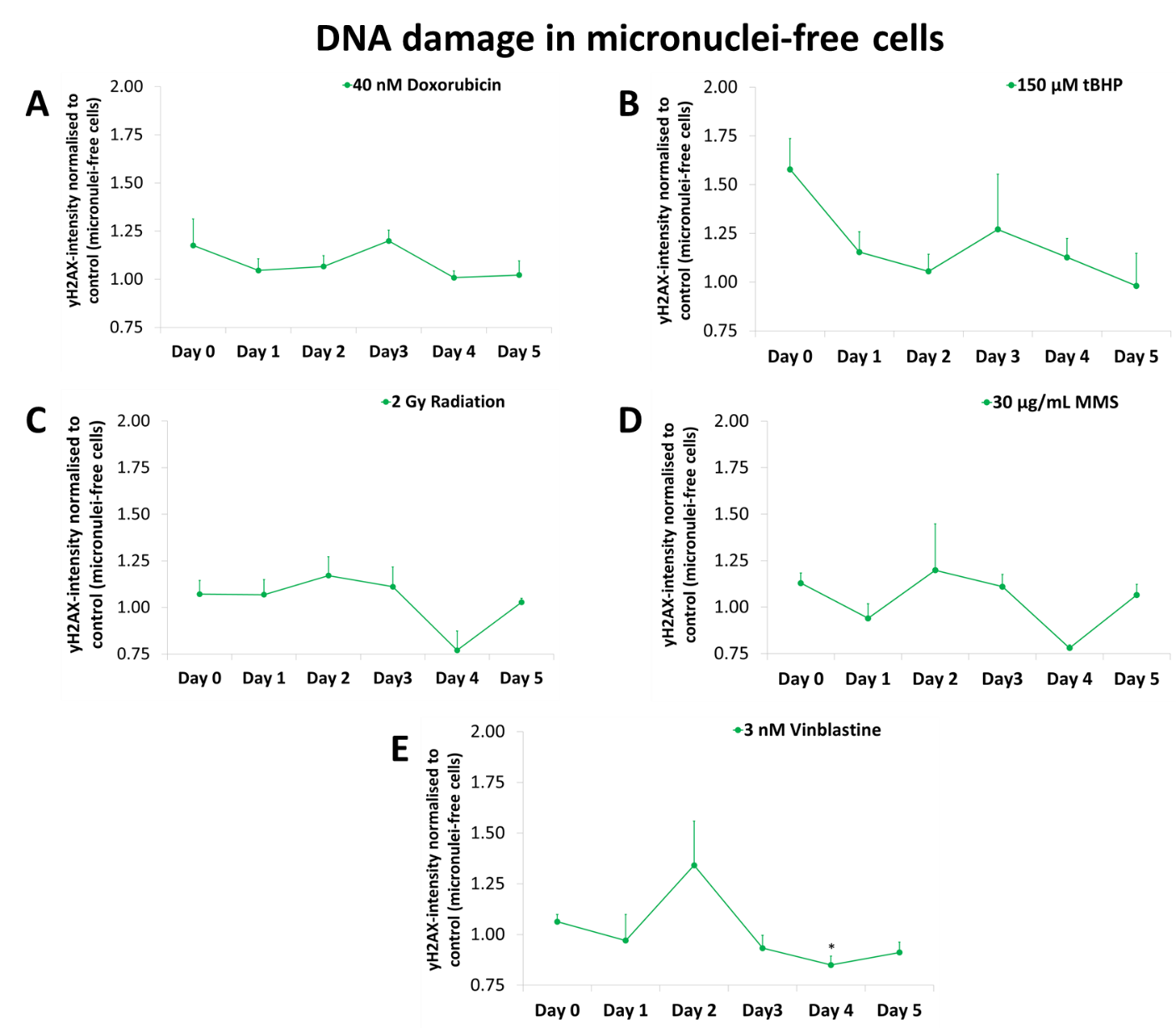


**Supplementary Fig. 7.** γH2AX-intensity normalised to main nuclei of micronuclei-free control cells in micronuclei-free cells of HeLa-H2B-GFP-cells treated with (A) 40 nM doxorubicin, (B) 150 µM tBHP, (C) 2 Gy radiation, (D) 30 µg/ml MMS and (E) 3 nM vinblastine. Green line indicate genotoxic agents, black line indicate respective solvent control cells (DMSO for doxorubicin and MMS experiments, water for tBHP, radiation and vinblastine experiments). All presented values are mean out of three experiments with standard error. Asterisks represent p<0.05 vs. control cells (t-test).


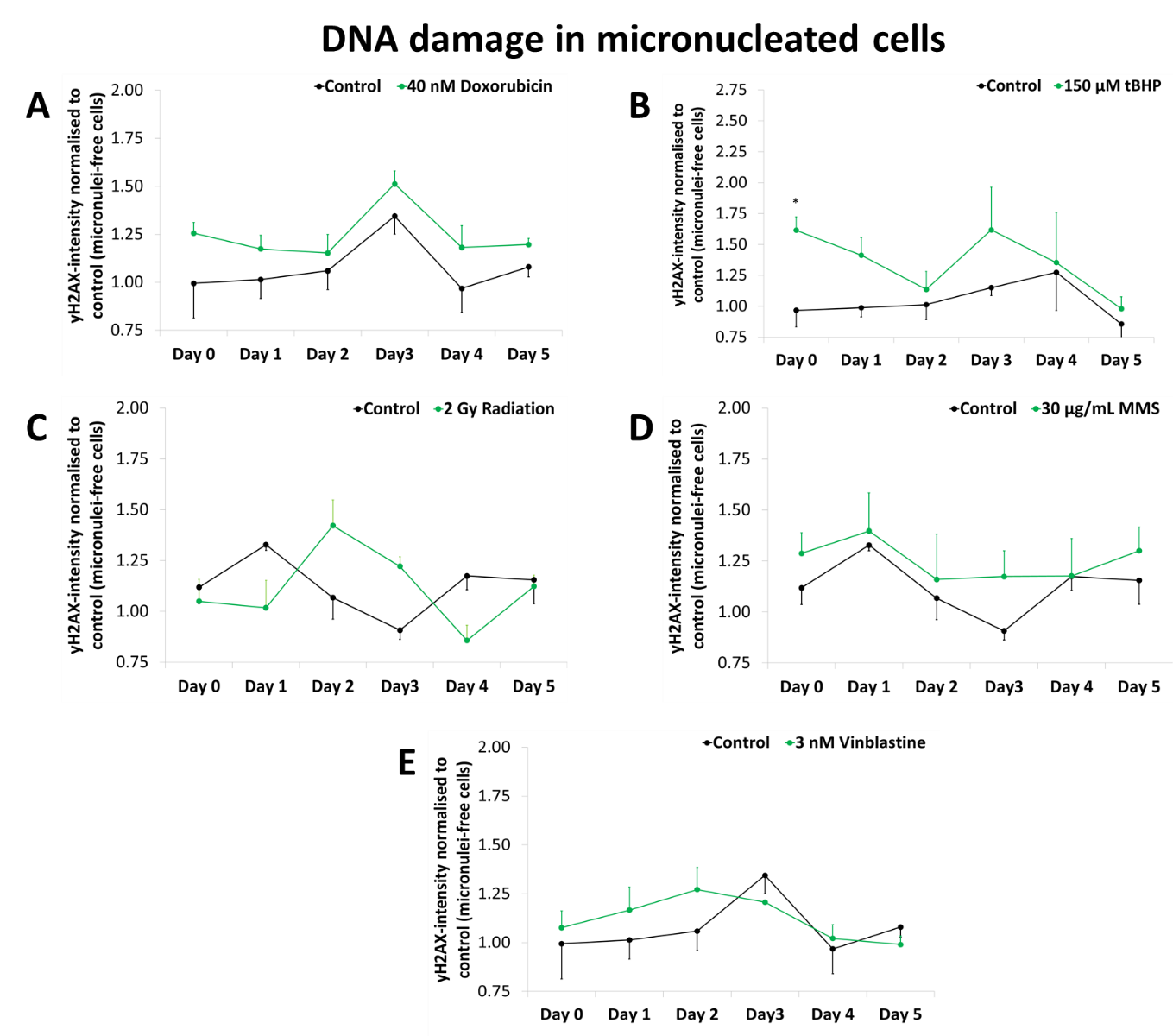


**Supplementary Fig. 8.** γH2AX-intensity normalised to main nuclei of micronuclei-free control cells in micronucleated cells of HeLa-H2B-GFP-cells treated with (A) 40 nM doxorubicin, (B) 150 µM tBHP, (C) 2 Gy radiation, (D) 30 µg/ml MMS and (E) 3 nM vinblastine. Green line indicate genotoxic agents, black line indicate respective solvent control cells (DMSO for doxorubicin and MMS experiments, water for tBHP, radiation and vinblastine experiments). All presented values are mean out of three experiments with standard error. Asterisk represent p<0.05 vs. control cells (t-test).
